# Single-cell chromatin landscapes associated with the burnt skin healing process in rats

**DOI:** 10.1038/s41597-025-04928-7

**Published:** 2025-04-16

**Authors:** Tao Wang, Bolin Zhu, Jianyu Lu, Xinya Guo, Ruikang Li, Yue Yuan, Junjie Chen, Xi Dai, Shuai Liu, Jiaxin Du, Xun Xu, Huan Liu, Xiaoyu Wei, Runzhi Huang, Shizhao Ji

**Affiliations:** 1 College of Life Sciences, University of Chinese Academy of Sciences, Beijing, 100049 China; 2 BGI Research, Hangzhou, 310030 China; 3 Department of Burn Surgery, The First Affiliated Hospital of Naval Medical University, Shanghai, 200433 China; 4 Department of Burn Surgery, the First Affiliated Hospital of Naval Medical University, No. 168 Changhai Road, Yangpu District, Shanghai, 200433 People’s Republic of China; 5 Key Laboratory of Resource Biology and Biotechnology in Western China, Ministry of Education, Provincial Key Laboratory of Biotechnology, College of Life Sciences, Northwest University, 229 Taibai North Road, Xi’an, Shaanxi 710069 China; 6 BGI Research, Shenzhen, 518083 China; 7 BGI College & Henan Institute of Medical and Pharmaceutical Sciences, Zhengzhou University, Zhengzhou, 450000 China; 8 State Key Laboratory of Agricultural Genomics, BGI-Shenzhen, Shenzhen, 518083 China; 9 BGI Life Science Joint Research Center, Northeast Forestry University, Harbin, 150040 China

**Keywords:** Computational biology and bioinformatics, Data acquisition

## Abstract

Thermal injuries represent one of the most severe forms of trauma to the human body, with a high annual incidence of burn victims globally. Skin regeneration and wound healing following thermal injury constitute a complex process involving various cell types and cytokine interactions. By Single-cell ATAC sequencing (scATAC-seq), we elucidated the molecular mechanisms underlying the dermal regeneration and healing processes following thermal injury in a rat model. Tissue samples were harvested for sequencing at predetermined intervals (0 h, 12 h, 24 h, 3 d, 7 d, 11 d, 15 d, and 19 d post-injury), yielding 28,179 high-quality single cells. Our comprehensive analysis revealed 28 distinct cell populations throughout the regenerative process, encompassing various subsets of keratinocytes, fibroblasts, and immune cells, exhibiting temporal heterogeneity across samples. Furthermore, we investigated the chromatin accessibility landscape of individual cell types and identified enriched transcription factor binding motifs, corroborating the robustness and validity of our data. Our dataset provides a valuable resource for further elucidation of burnt skin regeneration and healing processes.

## Background & Summary

The skin represents the largest and one of the most complex organ systems in mammals. It serves as a protective barrier lining the external surfaces of the mammalian body, shielding organisms from environmental insults^1^. Mammalian skin is a double-layered structure consisting of the epidermis and dermis and is characterized by a heterogeneous cellular composition that reflects its multifaceted functions and intricate organization^2^. Globally, many individuals sustain thermal injuries to the skin annually. The underlying cellular and molecular mechanisms of this process remain to be fully elucidated. Following burn injury, the healing process of damaged skin can be divided into three primary phases: (1) inflammatory, (2) proliferative, and (3) remodelling^3,4^. During these stages of burn wound healing, multiple cell types and cytokines interact in a coordinated manner to promote tissue regeneration and repair^3,5^. However, the transcription factors and epigenetic regulatory mechanisms underlying this process remain incompletely understood.

Single-cell assay for transposase-accessible chromatin sequencing (scATAC-seq) is a cutting-edge technology that has garnered substantial attention in recent years^6^. Compared to single-cell RNA sequencing (scRNA-seq), scATAC-seq can better capture subtle epigenetic regulatory variations among individual cells. This technique is particularly efficacious in elucidating transcriptional regulatory networks within cellular populations^7^. While numerous studies have employed scRNA-seq technology to investigate skin regeneration and wound healing processes^8–12^, research utilizing single-cell ATAC sequencing to decipher relevant DNA regulatory elements remains comparatively limited. While transcriptomic profiling enables the elucidation of functionally distinct cell populations within complex tissues, the integration of epigenetic data offers a more comprehensive understanding of the regulatory mechanisms governing these expression profiles and their maintenance^13,14^. The latter approach is critical for a comprehensive analysis of burn wound healing mechanisms, offering unique insights into the epigenetic landscape of the cellular populations involved in tissue repair.

In this study, we utilized scATAC-seq technology to elucidate the regeneration and healing process of rat skin following thermal injury. Tissue samples were collected for sequencing at eight time points post-injury: 0 h, 12 h, 24 h, 3 days, 7 days, 11 days, 15 days, and 19 days. Our findings reveal dynamic alterations in the cellular composition of the injured rat dermis and epidermis, as well as changes in the chromatin accessibility of specific cell types. In summary, our research provides a valuable resource for deciphering epigenetic regulation during the skin regeneration process following burn injuries.

## Methods

### Animal experiments and tissue collection

All animal experiments were conducted in accordance with protocols approved by the Department of Burn Surgery at the First Affiliated Hospital of Naval Medical University, Shanghai, China. Eight-week-old male Sprague‒Dawley rats (250–300 g) were utilized. Prior to the procedure, the rats were depilated to ensure complete exposure of the dorsal skin. On the day of the experiment, the rats were anaesthetized with isoflurane and placed on a heated pad with their head and limbs secured to facilitate dorsal skin exposure. A sponge pad was preheated to approximately 100 °C in boiling water for more than 10 minutes and then applied to the dorsal skin for 10 seconds to induce a burn. After the burn, the area was covered with oil gauze and secured with a pressure bandage. Tissue samples from the burn sites were collected at the following intervals: 0 hours, 12 hours, 24 hours, 3 days, 7 days, 11 days, 15 days, and 19 days post-burn. The samples were excised and prepared for single-cell ATAC-seq to assess local and systemic physiological, pathological, and inflammatory responses.

### Nuclear isolation from rat skin tissue

Nuclei were isolated from rat skin tissue for single-cell ATAC-seq using a refined enzymatic digestion and mechanical extraction approach^15^. Fresh skin samples, with a total area of approximately 1 cm², were first rinsed in PBS to remove visible fat and muscle debris. The cleaned tissue was then minced into small fragments using sterile surgical scissors and transferred to a 2 mL centrifuge tube. The tissue fragments were enzymatically digested by adding 1 mL of enzyme solution 1 (0.1% collagenase and 0.01% DNase) and incubating at 37 °C with gentle agitation (130 rpm) for 25 minutes. After digestion, the sample was centrifuged at 500 × g for 5 minutes, and the supernatant was discarded. The resulting pellet was resuspended in 10 mL of enzyme solution 2 (0.1% collagenase) and further incubated at 37 °C with agitation for an additional 60 minutes. The digested tissue suspension was then filtered through a 70 µm cell strainer to remove undigested fragments and washed with 2% FBS in DPBS. The suspension was further filtered through a 30 µm cell strainer. The filtered cell suspension was centrifuged at 300 × g for 5 minutes at 4 °C, and the pellet was resuspended in 1 mL of PBS containing 0.04% BSA. The cell viability and concentration were determined using trypan blue exclusion or an automated cell counter. The prepared nuclei were then utilized for scATAC-seq library preparation, ensuring high-quality samples for accurate chromatin accessibility analysis.

### scATAC-seq library preparation and sequencing

For the preparation of single-cell ATAC-seq libraries, we employed the DNBelab C Series Single-Cell ATAC Library Prep Set (MGI, H-020-000747-00)^16^. To investigate chromatin accessibility changes following burn injury in rats, we generated a total of 19 libraries. Specifically, 3 libraries were prepared from samples collected at 0 hours post-burn; 2 libraries were generated for each of the time points at 12 hours, 24 hours, 3 days, 7 days, 11 days, and 19 days post-burn; and 4 libraries were prepared for samples collected at 15 days post-burn. The barcoded scATAC-seq libraries were generated from transposed single-cell suspensions. In summary, the protocol involved droplet encapsulation, preamplification, emulsion breakage, collection of capture beads, DNA amplification, and purification. Afterwards, indexed sequencing libraries were prepared according to the manufacturer’s instructions. The concentrations of the sequencing libraries were measured via a Qubit ssDNA Assay Kit (Thermo Fisher Scientific). Sequencing was performed on the MGI DNBSEQ-T1 platform, with read lengths of 115 bp for read 1, 69 bp for read 2, and 10 bp for the sample index, following the sequencing scheme of the China National GenBank (CNGB)^17^.

### Preprocessing of scATAC-seq data

The preprocessing of scATAC-seq data followed a series of well-defined steps. First, raw sequencing reads were demultiplexed to separate insertions and barcodes. The reads were then filtered using PISA (version 1.1)^18^, with a minimum sequencing quality threshold of 20. PISA is available at https://github.com/shiquan/PISA. The sequencing reports for the scATAC-seq datasets are detailed in Table 1. After filtering, the reads were aligned to the rat reference genome (mRatBN7.2) using BWA (version 0.7.17-r1188)^19^. The aligned BAM files were subsequently processed using bap2 (version 0.6.2) to group barcodes belonging to the same cell for downstream analysis^20^. This preprocessing workflow ensured high-quality data for further chromatin accessibility analysis.

**Table 1:** Each library metadata and mapping statistics.

	total_reads	pass_reads	number of cells passed QC	mean fragments per cell	mean TSS Enrichment
control	437,685,922	389,505,620	6,338	13956.39	12.97
burn-0h-1	552,643,237	484,620,787	786	14577.59	8.90
burn-0h-2	607,214,163	530,005,216	491	18293.98	8.56
burn-12h-1	801,545,511	710,192,772	128	6493.26	6.09
burn-12h-2	621,626,782	550,538,857	94	5632.89	5.83
burn-24h-1	1,655,127,472	1,459,956,275	548	7407.06	7.10
burn-24h-2	859,138,439	758,020,281	298	4053.62	6.42
burn-3D-1	410,784,378	361,408,254	1,426	6685.67	9.22
burn-3D-2	601,389,487	528,758,313	145	14750.50	7.66
burn-7D-1	327,975,388	285,789,352	959	15224.55	10.49
burn-7D-2	568,409,773	493,279,946	305	33090.22	9.96
burn-15D-1	476,831,820	414,912,997	4,946	9846.85	11.47
burn-11D-1	625,933,306	546,478,639	2,623	13808.93	12.45
burn-11D-2	479,000,183	420,501,537	2,065	10196.34	12.28
burn-15D-2	425,694,294	370,896,942	3,431	10115.62	11.56
burn-15D-3	515,115,975	450,509,546	536	6084.26	9.71
burn-15D-4	636,273,182	552,775,087	120	9848.68	7.61
burn-19D-1	377,784,204	334,476,078	1,404	7156.74	12.86
burn-19D-2	680,501,343	602,208,928	1,536	12026.49	11.42
Total/Average	11,660,674,859	10,244,835,427	28,179	11539.45	9.61

### Quality control for scATAC-seq data

We used the AchR (version 1.0.2)^21^ package to create arrow files and ArchR projects via the ‘createArrowFiles’ and ‘ArchRProject’ functions. The doublet scores of the cells were calculated by the ‘addDoubletScores’ function. In this way, low-quality cells were filtered with the same criteria for all scATAC-seq libraries: unique nuclear fragments (nFrags) >1000 and an enrichment score of the transcriptional start site (TSS) >4. We also filtered doublets with parameters of filterRatio = 1 by the ‘filterDoublets’ function.

### Dimension reduction and scATAC cell clustering

The ‘addIterativeLSI’ function in ArchR^21^ was used to implement the iterative latent semantic indexing (LSI) dimensionality reduction. The ‘addClusters’ function was subsequently used to cluster cells via the default Seurat^22^ graph clustering approach in this reduced-dimensional subspace. We also implemented uniform manifold approximation and projection (UMAP) in this subspace to visualize all the cells via the ‘addUMAP’ function.

### Batch effect correction and library integration

We tried a batch effect correction tool called Harmony with the ‘addHarmony’ function on our scATAC-seq data to correct for batch effects. However, no significant batch effects were detected. Therefore, we directly merged all the libraries into one ArchR^21^ object for downstream analysis.

### Cell type annotation and subpopulation identification

Using the ‘getMarkerFeatures’ function and the ‘plotEmbedding’ function with default parameters, we obtained and visualized the marker genes for each cell cluster. We accurately annotated each cell type manually on the basis of the cell markers summarized in previous studies. To demonstrate the high quality of the data, we separately filter some of the fibroblasts and keratinocytes to find subclusters via the ‘addClusters’ function, with ‘resolution = 0.15’.

### Integration of scRNA-seq data and scATAC-seq data

We first obtained single-cell RNA-seq data from radiation-induced skin injury (GSE193564)^23^. We then converted our burnt skin scATAC-seq ArchR objects into Seurat objects using gene scores. We subsequently integrated the publicly available scRNA-seq datasets and our scATAC-seq data using Seurat, employing the anchor-based CCA integration approach for multimodal integration.

### Cell type-specific peak calling

Before calling the cell type-specific peak, we made pseudobulk replicates by using the ‘addGroupCoverages’ function with the key parameter ‘groupBy = celltype’. With cell type pseudobulk replicates created, we used MASC2^24^ to call peaks for each cell type via the ‘addReproduciblePeakSet’ function with the following parameters: groupBy = cluster_name, pathToMacs2 = pathToMacs2, genomeAnnotation = genomeAnnotation, geneAnnotation = geneAnnotation, genomeSize = 2647899415. The marker peaks of each cell type were identified via the ‘addMarkerFeatures’ function with useMatrix = “PeakMatrix”.

### Motif enrichment for cell types

We obtained the mouse motif annotation database “mouse_pwms_v1” from the chromVARmotifs package (https://github.com/GreenleafLab/chromVARmotifs). Then, we add motif annotation to the ArchR object via the ‘addMotifAnnotations’ function. Next, motif enrichment analysis was executed by using the ‘peakAnnoEnrichment’ function on the marker peaks of each cell type obtained previously.

### Temporal profiling of chromatin accessibility dynamics

To elucidate the temporal changes in chromatin accessibility, we extracted the accessibility matrices of peaks from monocytes and vascular endothelial cells. For each peak, we calculated the average accessibility matrix across different time points. We employed the Mfuzz^25^ R package (version 2.58.0) to perform C-means clustering on the accessible peaks in both monocytes and vascular endothelial cells. We subsequently utilized the clusterProfiler^26^ R package (version 4.6.0) to perform Gene Ontology (GO) functional enrichment analysis for different peak clusters produced by Mfuzz.

### TF footprint analysis

We used the ‘getPositions’ function to obtain the genome positions of the cell type-specific motifs. With group coverages calculated previously, we computed footprints for marker motifs via the ‘getFootprints’ function and visualized them via the ‘plotFootprints’ function.

## Data Records

The chromatin accessibility landscapes of burnt rat skin at different healing times were profiled via the scATAC-seq technique. The panoramic landscape consisting of 28179 high-quality single cells provides a valuable resource for exploring the epigenetic regulation of the burnt skin healing process at the single-cell level. All related raw fastq files have been submitted to the CNGB Nucleotide Sequence Archive (CNSA) under accession number CNP0006144^27^. Moreover, the cell type-peak matrices and other metadata have been stored in Figshare (https://figshare.com/s/f21a969a6e598f8d3551)^28^.

## Technical Validation

### Data quality control for scATAC-seq

**Fig. 1:**
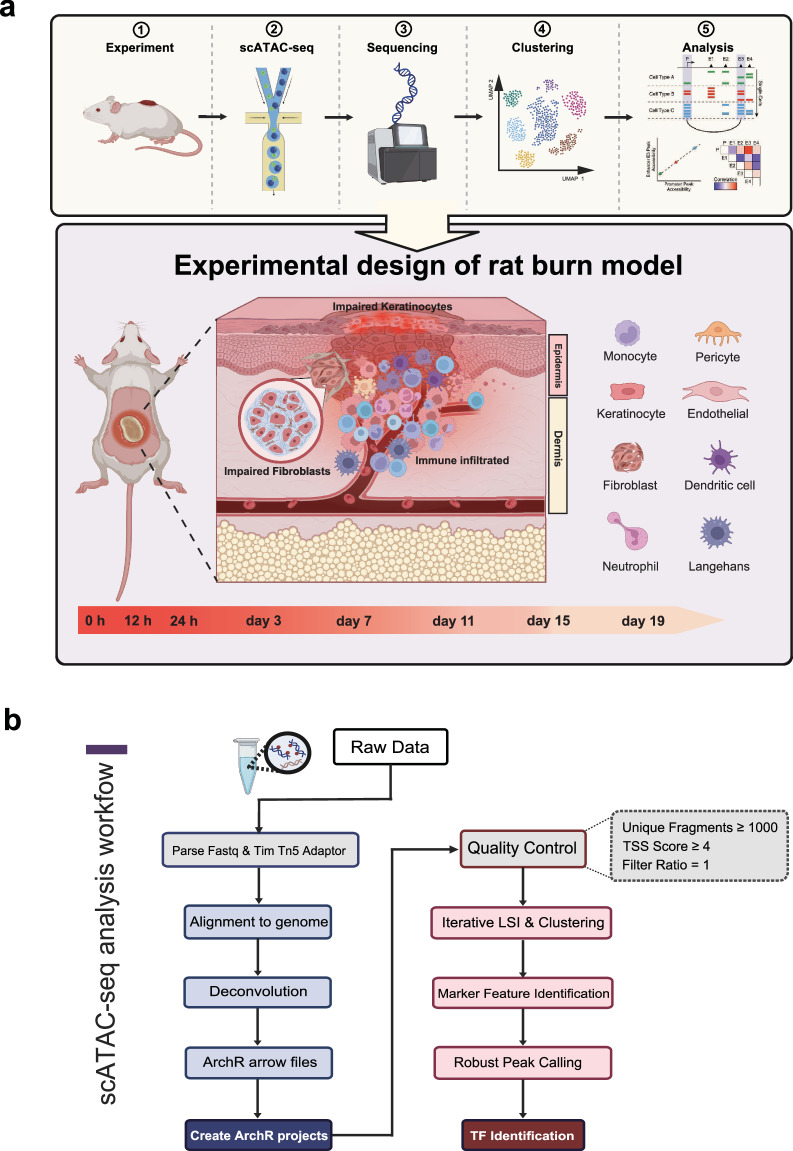
Overview of the experimental design and data analysis workflow for scATAC-seq of burnt rat skin. (**a**) Schematic representation of the experimental timeline and sample collection. Burn injury was induced, and skin samples were harvested at 0 h, 12 h, 24 h, 3 days, 7 days, 11 days, 15 days, and 19 days post-injury for single-cell assays for transposase-accessible chromatin sequencing (scATAC-seq). (**b**) scATAC-seq analysis workflow.

**Fig. 2:**
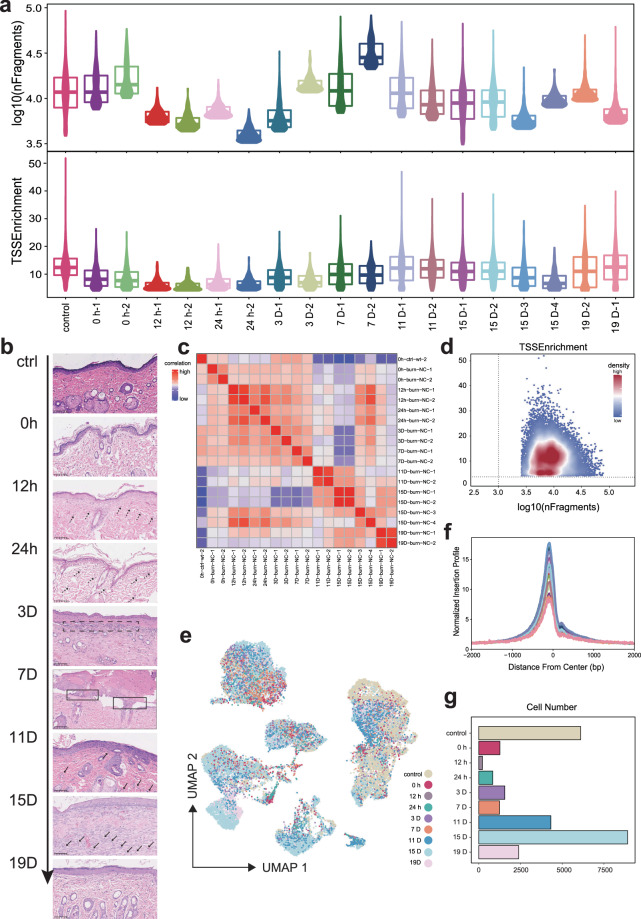
Quality control and preprocessing of scATAC-seq data from burnt rat skin. (**a**) Violin plot showing the distribution of log_10_(nFragments) and TSS enrichment scores for each library, with libraries grouped by burn injury status and time points. (**b**) H&E-stained sections of rat skin under normal conditions and at various time points post-burn (0–19 days) (×20). Immune cell infiltration is observed in the early burn stage (≤3D), followed by extensive proliferation of fibroblasts and parenchymal cells (7D–11D) and extracellular matrix remodelling (15D–19D). (**c**) Heatmap showing the correlations between different libraries. (**d**) Scatter plot comparing log_10_(nFragments) and TSS enrichment scores, revealing the quality of the data. (**e**) UMAP plot visualizing the distribution of cells across different time points post-burn injury, with each dot representing a single cell and the colour indicating the time point. (**f**) Transcription start site (TSS) enrichment profiles for each library. (**g**) Bar chart showing the number of cells captured at each time point.

In this study, single-cell nuclei were extracted from rat skin at 0 h, 12 h, 24 h, and 3, 7, 11, 15, and 19 days after burn injury (see Methods). H&E-stained sections of burn skin samples at each time point revealed distinct skin structure statuses. Round-shaped cells (indicated by dashed arrows at 12 h and 24 h and dashed boxes at 3D) were identified as myeloid cells. These cells first emerged at 12 h post-injury and then progressively increased, peaking at 3D. Between 7D and 15D, elongated spindle-shaped cells demarcated by solid boxes and solid arrows were recognized as fibroblasts, which exhibited extensive proliferation to repopulate the wound. Although wound closure was observed from 15D to 19D, histological analysis revealed incomplete restoration of native skin architecture. (Figs. 1a, 2b). At each time point, two single-cell ATAC-seq libraries, both derived from the same individual rat, were generated. However, at the Day 15 time point, four distinct libraries were constructed using biological replicates from two rats: libraries 15D-1 and 15D-2 originated from one rat, whereas libraries 15D-3 and 15D-4 were prepared from a second rat. The total number of raw reads from all the libraries was 11,660,674,859. In addition, 10,244,835,427 passed quality control (Table 1). Next, the raw fastq data were processed via a standardized pipeline including alignment to the genome and ArchR^21^ downstream analysis (Fig. 1b).

To ensure that each cell was of high quality, we calculated transcriptional start site (TSS) enrichment scores, unique fragments, and the number of fragments of each cell. After filtering low-quality cells with the criterion (TSS > 4, nFrags > 1000, Doublet filterRatio = 1) (see Methods), we reserved a total of 28179 high-quality cells with chromatin accessibility profiles. The number of cells retained at each time point is shown in a bar plot (Fig. 2g). Among these remaining cells, the mean number of fragments per cell and the mean TSS enrichment were 11,539.45 and 9.60672, respectively (Table 1) (Fig. 2a,d,f). We also calculated the correlation of each scATAC-seq library data expression profile on the basis of the peak accessibility matrix (Fig. 2c). The UMAP colour at different time points during skin wound healing revealed diverse cell type distributions during the healing process (Fig. 2e).

### Cell type identification by chromatin accessibility

**Fig. 3:**
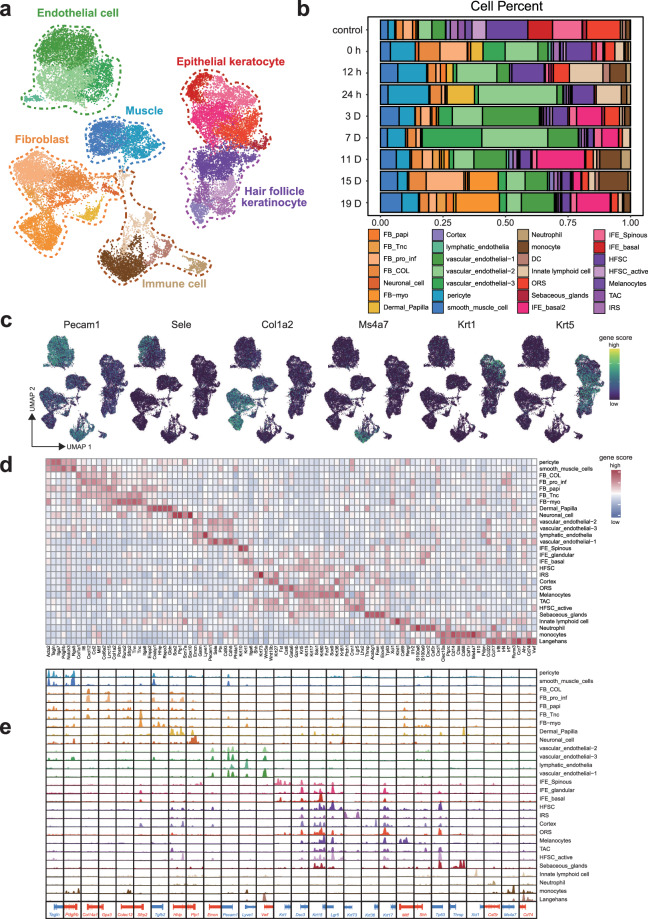
Identification of major cell types through scATAC-seq analysis during the rat burnt skin healing process. (**a**) UMAP plot displaying the separation of six primary cell types on the basis of their chromatin accessibility profiles. Each cluster is labelled with its corresponding cell type. (**b**) Stacked bar graph showing the proportion of each cell type across all samples. (**c**) UMAP visualization of the cell type-specific gene activity score. (**d**) Heatmap of cell type-specific marker gene activity scores. (**e**) Genome browser view of the aggregated scATAC-seq chromatin accessibility profiles of housekeeping genes and cell type-specific gene loci.

After that, all cells collected from the skin at all time points of burn healing were subjected to unsupervised clustering on the basis of gene score values. On the basis of the differential chromatin accessibility within some clusters, we implemented cell-subpopulation clustering in one keratinocyte cluster and two fibroblast clusters. Finally, we defined 28 cell clusters, including endothelial cells, fibroblasts, muscle cells, immune cells, epidermal keratinocytes, and hair follicle keratinocytes. Among all keratinocyte subtypes, three subtypes of epidermal keratinocytes (IFE_basel1 (Krt5, Krt15), IFE_basel2 (Prss27, S100a8), and IFE_spinous (Krt1, Krt10)) and eight subtypes of cells in hair follicles (HFSC (Lgr5, Lhx2), IRS (Krt73, Shh, Krt27), cortex (Krt36, Krt27, Krt81), ORS (Fst, Sdc1, Krt17, Gata6), melanocytes (Mitf, Mlana), TAC (Shh, Wnt10b, Krt27), HFSC_activate (Lgr5, Tp63), and sebaceous glands (Thrsp, Acsbg1, Elovl5)) were identified. Among all fibroblast subtypes, the dermal papilla expresses the most unique marker genes (Enpp2, Crabp1, Hhip, Rspo3, and Gldn). Our data revealed that myofibroblasts expressed the following markers: Postn, Runx2, Sfrap2 and Tnn. Other fibroblasts were divided into FB_COL, which more highly expresses Col1a1 and Col1a2; FB_pro_inf, which more highly expresses some inflammation-related genes (Il6, Cxcl12, and Ccl2); FB_papi, which more highly expresses Enpp2, Crabp1, Cspg4 and Colec12; and FB_Tnc, which more highly expresses Tnc and other myofibroblast marker genes. We also detected 4 immune cell populations, namely, neutrophils (S100a8, S100a9, and Cxcr2), monocytes (Cd14, Ctss, Ptprc, and Cd68), dendritic cells (DCs) (Cd74, Irf8, Irf4, and Irf7) and innate lymphoid cells (Xcl1, Klrd1, Cpa3, and Cd69). The muscle cells consisted of two cell types: pericytes (Acta2, Tagln) and smooth muscle cells (Notch3, Rgs5). The endothelial cells were divided into 4 groups: lymphatic endothelial cells (Lyve1, Pdpn) and 3 types of vascular endothelial cells. Interestingly, we also detected a neural cell type (Scn7a, Plp1) (Fig. 3d). The chromatin accessibility of these genes is also shown (Fig. 3g). These cells constitute the microenvironment during the healing process of rat skin burn injuries.

By comparing the proportions of cell types at different healing time points, we determined that keratinocytes, including epithelial keratinocytes and hair follicle keratinocytes, were the most abundant in normal samples (Fig. 3b). The number of keratinocytes, especially epidermal keratinocytes, was significantly decreased after burn injury, indicating keratinocyte apoptosis. Moreover, the proportions of immune cells, such as neutrophils, monocytes, and innate lymphoid cells, increased significantly at 0 h, 12 h, and 24 h after burn injury, indicating immune infiltration in burn wounds (Fig. 3b).

### Integrative analysis of public rat injured skin scRNA-seq datasets

To validate the reliability of our scATAC-seq data and reveal the deeper biological importance, we performed integrative analysis between our scATAC-seq dataset and publicly available scRNA-seq data of radiation-induced skin injuries (spanning 0, 7, 14, and 28 days post-injury). We reanalyzed and annotated the scRNA-seq data on the basis of the marker genes reported in the original publications. (Supplementary Fig. 1c). The integration of multi-omics data revealed a high degree of correlation between cell types in our scATAC-seq and public scRNA-seq data. (Supplementary Fig. 1a,b). Notably, our chromatin accessibility profiles enabled higher-resolution cellular clustering (Supplementary Fig. 1d), exemplified by the identification of six fibroblast subtypes, including the presence of myofibroblasts in the postrepair period of burned skin and distinct subpopulations of keratinocytes that constitute the epidermis and hair follicles, respectively. These findings underscore both the superior discriminative power of scATAC-seq in cell type delineation and the comprehensive nature of our dataset.

### Dynamic changes in chromatin accessibility hinder the skin healing process

**Fig. 4:**
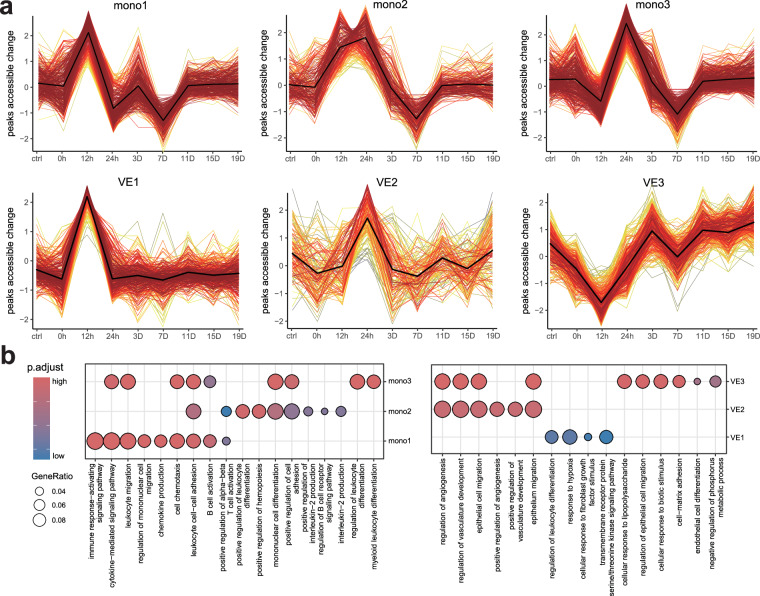
Identification of temporal chromatin accessibility through the burnt skin healing process. (**a**) Six key peak clusters identified by Mfuzz: Mono1/2/3 (monocytes) and VE1/2/3 (vascular endothelial cells). (**b**) GO functional enrichment for peak clusters in monocytes and vascular endothelial cells.

Given that our time series data comprehensively captured the early immune response and the vascularization process during the skin healing process, we aimed to describe the changes in gene accessibility and the key molecular mechanisms involved in these processes over time. Consequently, we extracted accessible peaks from monocytes and vascular endothelial cells and analysed the temporal trends of gene expression accessibility using the Mfuzz, which employs a fuzzy c-means clustering approach. In the analysis of monocytes, we identified three key peak clusters, Mono1 (n = 251), Mono2 (n = 180), and Mono3 (n = 321), which exhibited high accessibility at 12 hours, 12–24 hours, and 24 hours post-injury, respectively. In vascular endothelial cells, VE1 (n = 267), VE2 (n = 122), and VE3 (n = 271) were highly accessible at 12 hours, 24 hours, and 3–19 days post-injury, respectively (Fig. 4a).

We performed GO functional enrichment analysis on the nearest genes corresponding to these accessible peaks. The results revealed that the functions influenced by the accessible peaks in Mono1 are associated primarily with immune response activation, chemokine production, and monocyte migration. These findings suggest that at 12 hours post-injury, monocytes are in a state of immune activation and migrate towards the wound. The accessible peaks in Mono2 mainly affect functions related to interleukin-2 production, as well as the activation of B cells, T cells, and monocyte differentiation. This finding indicates that between 12 and 24 hours post-injury, monocytes begin to further differentiate into mature monocyte subtypes and release cytokines such as interleukin-2 to influence the activation of other lymphoid cells. The accessible peaks in Mono3 primarily influence the differentiation of leukocytes and myeloid cells, as well as the accessibility of genes related to chemotaxis and cell adhesion. They regulate the activity of immune cells and promote signalling interactions with other cells (Fig. 4b).

The accessible peaks in VE1 primarily influenced genes associated with hypoxia, as well as the regulation of leukocyte differentiation. These findings indicate that at 12 hours post-injury, vascular endothelial cells adapt to environmental changes and secrete cytokines that influence immune regulation. The peaks in VE2 mainly affected genes related to angiogenesis regulation and development, suggesting that by 24 hours post-injury, vascular endothelial cells have already opened up chromatin regions associated with vascularization to promote tissue repair. The peaks in VE3, in addition to influencing genes related to endothelial cell differentiation, predominantly affected genes associated with cell-matrix adhesion and the response to biotic stimuli. This finding suggests that from 3 to 19 days post-injury, vascular endothelial cells enter a state of endothelial differentiation and interact with the external environment through cell adhesion and lipopolysaccharides (Fig. 4b).

### Cell type-specific peak calling and motif enrichment

**Fig. 5:**
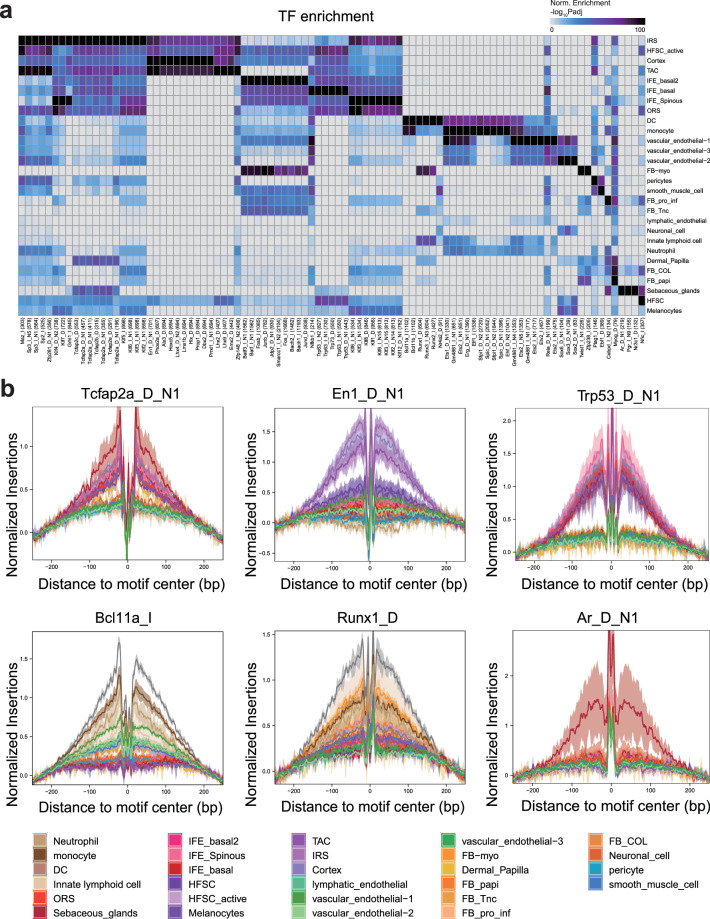
Transcription factor-binding site enrichment and motif activity profiling in the rat burnt skin healing process. (**a**) Heatmap depicting the enrichment of transcription factors (TFs) across various cell types. (**b**) Footprint profiles for selected TFs in different cell types.

For each cell type, we performed pseudobulk replicates and peak calling, ultimately obtaining a total of 748906 peak sets. We also identified cell type markers and implemented motif enrichment on these peaks to identify potential transcription factors (TFs) associated with these open chromatin regions. Interestingly, Kruppel family^29,30^ (Klf4, Klf7) and AP-2 family^31,32^ (Tcfap2a, Tcfap2b, and Tcfap2c) genes, such as IRS and IFE_Spinous, were enriched in keratinocytes. En1^33^, Hoxc5^34^ and Alx3^35^ were specifically enriched in hair follicle keratinocytes, such as those in the cortex and TAC. In addition, the p53^36^ family (Trp53, Trp63, Trp73) was more highly enriched in epidermal keratinocytes. Dendritic cells were enriched for motifs of the Bcl11^37,38^ family (Bcl11a, Bcl11b) and the Runx^37^ family (Runx1, Runx2, Runx3). Sebaceous glands were enriched for the Ar^39^, Pgr and Nr3c1 motifs (Fig. 5a). To further confirm the dependability of these motifs, transcription factor (TF) footprinting was performed, which confirmed that the motif bound to the TFs. Indeed, the results were consistent with those of the motif enrichment analysis (Fig. 5b).

In summary, the results provide high-quality data that we generated to profile the epigenetic process of burn wound healing.

## Usage Notes

The pipeline of scATAC-seq data analysis, including read quality control, read mapping, read counting, low-quality cell removal, dimension reduction, unsupervised clustering, peak calling and motif enrichment, was run on the Linux operating system. All source R codes used for data analysis are provided online.

## Supplementary information

Supplementary Figure 1

## Data Availability

All the codes for scATAC-seq downstream analysis in this paper were shared online. (https://figshare.com/s/f21a969a6e598f8d3551) and (https://github.com/BGI-TaoWang/Rat-burnt-skin-scATAC-seq-atlas).
