# Supplementary material for: Single-cell chromatin landscapes associated with the burnt skin healing process in rats
Source: Sci Data. 2025 Apr 16;12:639. doi: 10.1038/s41597-025-04928-7 (PMC12003776; doi:10.1038/s41597-025-04928-7)
Supplement: Supplementary file 1 — Supplementary Figure 1 [file 41597_2025_4928_MOESM1_ESM.pdf]

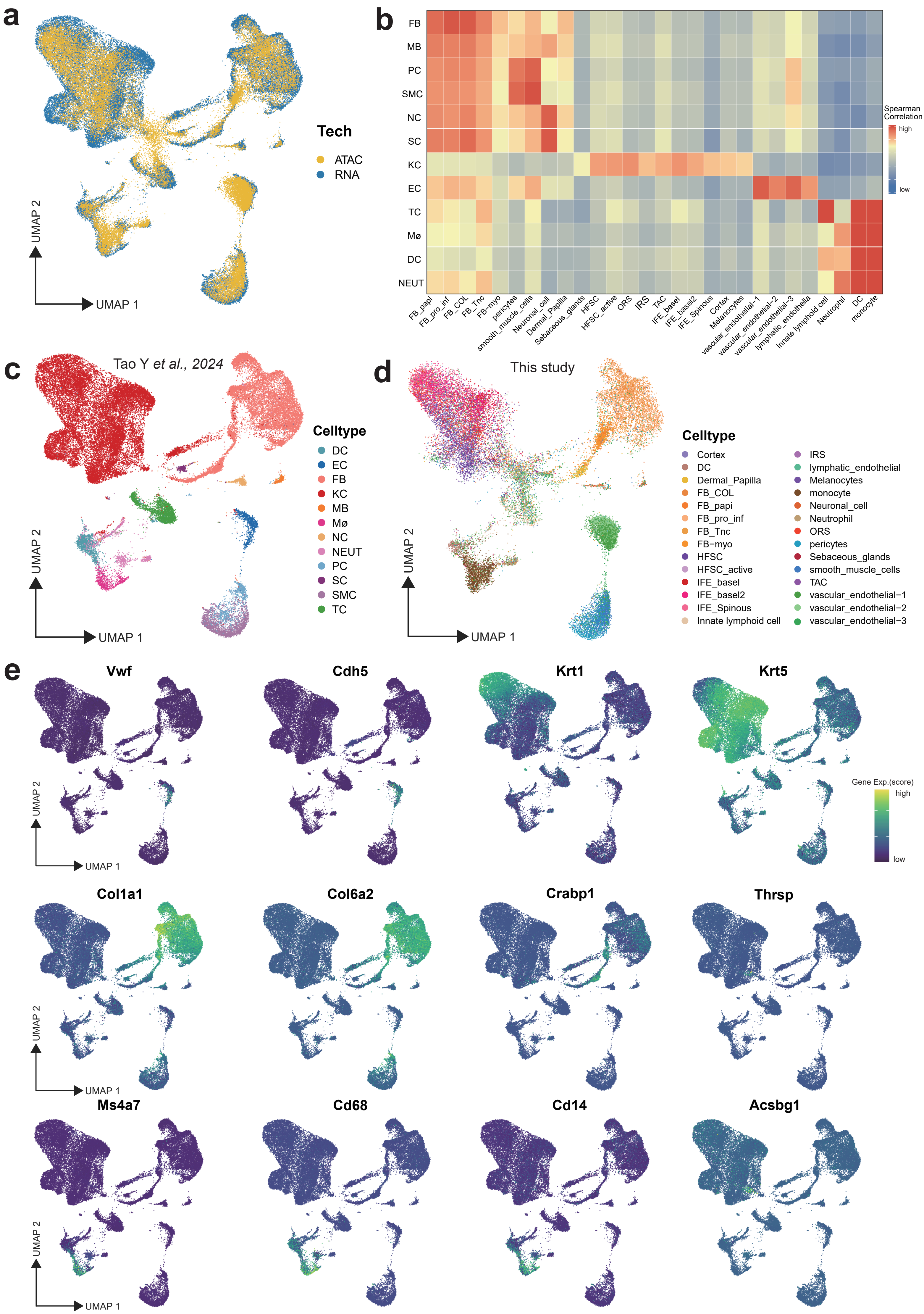
a
Tech
ATAC
RNA
UMAP 2
UMAP 1
b
FB
MB
PC
SMC
NC
SC
KC
EC
TC
Mø
DC
NEUT
Spearman Correlation
high
low
FB_papi
FB_pro_inf
FB_COL
FB_Tnc
FB-myo
pericytes
smooth_muscle_cells
Neuronal_cell
Dermal_Papilla
Sebaceous_glands
HFSC
HFSC_active
ORS
IRS
TAC
IFE_basel
IFE_basel2
IFE_Spinous
Cortex
Melanocytes
vascular_endothelial-1
vascular_endothelial-2
vascular_endothelial-3
lymphatic_endothelia
Innate lymphoid cell
Neutrophil
DC
monocyte
c
Tao Y et al., 2024
Celltype
DC
EC
FB
KC
MB
Mø
NC
NEUT
PC
SC
SMC
TC
UMAP 2
UMAP 1
d
This study
Celltype
Cortex
DC
Dermal_Papilla
FB_COL
FB_papi
FB_pro_inf
FB_Tnc
FB−myo
HFSC
HFSC_active
IFE_basel
IFE_basel2
IFE_Spinous
Innate lymphoid cell
IRS
lymphatic_endothelial
Melanocytes
monocyte
Neuronal_cell
Neutrophil
ORS
pericytes
Sebaceous_glands
smooth_muscle_cells
TAC
vascular_endothelial−1
vascular_endothelial−2
vascular_endothelial−3
UMAP 2
UMAP 1
e
Vwf
Cdh5
Krt1
Krt5
Gene Exp.(score)
high
low
Col1a1
Col6a2
Crabp1
Thrsp
Ms4a7
Cd68
Cd14
Acsbg1
UMAP 2
UMAP 1

Supplementary Figure 1. Integration of our scATAC-seq dataset with the scRNA-seq dataset of rat skin with radiation injury by Tao Y et al.

(a) UMAP displays cell clusters derived from two datasets. (b) Heatmap shows the correlation between our scATAC dataset and the public scRNA dataset, with scATAC cell types on the x-axis and scRNA cell types on the y-axis. (c) UMAP shows cell clusters from public scRNA-seq. (d) UMAP shows cell clusters from public scATAC-seq. (e) FeaturePlot shows marker gene expression in the public scRNA-seq dataset.
